# A new method for measuring lung deposition efficiency of airborne nanoparticles in a single breath

**DOI:** 10.1038/srep36147

**Published:** 2016-11-07

**Authors:** Jonas K. F. Jakobsson, Johan Hedlund, John Kumlin, Per Wollmer, Jakob Löndahl

**Affiliations:** 1 Div. of Ergonomics and Aerosol Technology (EAT), Dep. of Design Sciences, Lund University, SE-221 00, Lund, Sweden; 2 NanoLund, Lund University, Box 118, 22100 Lund, Sweden; 3 Dept. of Translational Medicine, Lund University, SE-221 00, Malmö, Sweden

## Abstract

Assessment of respiratory tract deposition of nanoparticles is a key link to understanding their health impacts. An instrument was developed to measure respiratory tract deposition of nanoparticles in a single breath. Monodisperse nanoparticles are generated, inhaled and sampled from a determined volumetric lung depth after a controlled residence time in the lung. The instrument was characterized for sensitivity to inter-subject variability, particle size (22, 50, 75 and 100 nm) and breath-holding time (3–20 s) in a group of seven healthy subjects. The measured particle recovery had an inter-subject variability 26–50 times larger than the measurement uncertainty and the results for various particle sizes and breath-holding times were in accordance with the theory for Brownian diffusion and values calculated from the Multiple-Path Particle Dosimetry model. The recovery was found to be determined by residence time and particle size, while respiratory flow-rate had minor importance in the studied range 1–10 L/s. The instrument will be used to investigate deposition of nanoparticles in patients with respiratory disease. The fast and precise measurement allows for both diagnostic applications, where the disease may be identified based on particle recovery, and for studies with controlled delivery of aerosol-based nanomedicine to specific regions of the lungs.

There is a need for efficient and precise methods for determination of respiratory tract deposition of inhaled airborne nanoparticles within pulmonary drug delivery, respiratory diagnostics and toxicology. The respiratory tract deposition of inhaled nanoparticles is a key link to understanding their health effects. In this paper, a method is presented to measure directly the deposition of nanoparticles in a controlled, low dose, single breath procedure.

To date only about 50 studies have been published that report experimental results for measuring respiratory tract deposition of particles <300 nm in human lungs^1^. Differences in methodology and small groups of subjects have contributed to significant divergence in the reported results^2^,^3^,^4^,^5^,^6^,^7^,^8^,^9^. Many of the developed methods also take considerable time and effort to execute, in some cases several hours of measurements and as a consequence the inhaled particle dose has been substantial^5^,^10^.

No system for direct measurements of lung deposition of nanoparticles is commercially available, and there is no consensus of a standard reference with which to compare the different techniques. There are, however, several commercially available devices for monitoring the concentration, and assumed exposure, of inhalable airborne nanoparticles based on a model of average respiratory tract deposition fractions for hydrophobic particles^1^. This approach provides estimations of exposures to airborne nanoparticles for an idealized normal case, but neither take into account the vast individual variations^11^ of anatomical and physiological features, nor the varying properties of real-world aerosol particles. A device for direct measurements of respiratory tract deposition of nanoparticles is of great importance to reach a better understanding of the mechanisms determining the real-world situation, taking these, and possible other previously unknown factors into account.

Several critical design aspects have been identified as potential sources of uncertainty in previous studies^1^ and should be taken into account. Most problems can be directly related to the aerosol source, the inhalation system or the particle detection system^1^. There is a lack of simple, robust and reliable particle generators producing hydrophobic nanoparticles suitable for inhalation studies. Particle losses in the instrument are unavoidable and have to be minimized and accounted for during measurements. Detection and precise monitoring of nanoparticle concentrations is challenging due to their limited light scattering properties and small mass. We intend to implement state-of–the-art technology to solve these issues. The nano aerosol will be generated by an electrospray followed by electrostatic size-selection. The particle detection will be performed by a condensation particle counter and particle losses in the instrument will be accounted for by careful characterisation and particle loss correction models.

The aim of this study is to design an instrument for direct measurement of lung deposition of nanoparticles and to investigate suitable operational parameters for measurements on human subjects. A second aim is to evaluate the performance of the instrument with regard to precision, sensitivity and reproducibility for different particle sizes and breathing conditions in a group of healthy volunteers. Putative applications for the technology includes controlled nanoparticle drug delivery, assessment of lung deposition in toxicological studies and diagnosis based on differences in deposition between normal and diseased lungs.

## Material and Methods

### Experimental setup

**Figure 1:**
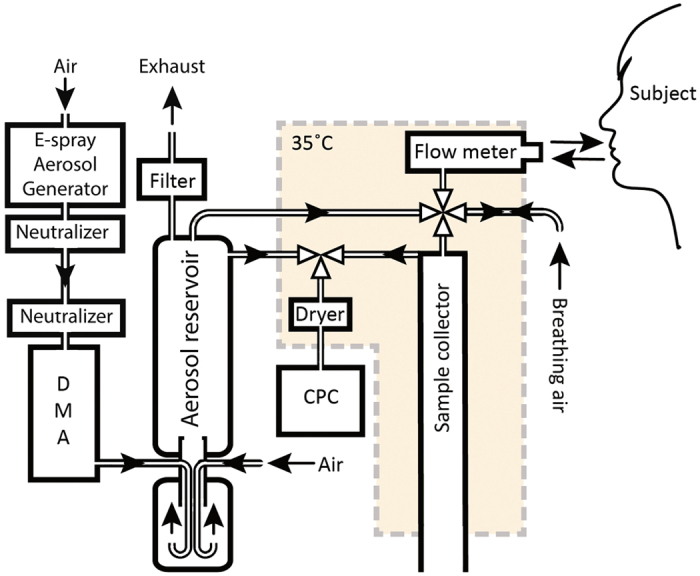
Schematic illustration of the instrument. The aerosol is produced with an electrospray aerosol generator (E-spray), size selected with a differential mobility analyser (DMA) and transferred into an aerosol reservoir where it is diluted and mixed with particle free air. Aerosol from the distal airspaces is sampled into a separate volume and the particle concentration in inhaled and exhaled aerosol is measured with a condensation particle counter (CPC).

As illustrated in Fig. 1, the instrument for measurement of particle deposition in the lungs consists of three main parts: 1) aerosol generation and conditioning, 2) inhalation system and 3) particle detection and analysis. The system is controlled by a computer with software written in LabView (LabView 10.0, National Instruments, US). Overall, the design follows principles previously described for respiratory tract deposition measurements^1^.

### Aerosol generation

The aerosol was generated by electrospraying polystyrene latex nanospheres (PSL) (Polymer Microsphere Suspension, Microgenics Corp, Fremont CA, US) with an electrospray aerosol generator (model 3480, TSI Inc., Shoreview, MN, US). The aerosol generator contains a built-in Po-210 neutralizer (P-2042 Nuclespot Alpha ionizer, NRDStaticControl, NY, US) at the aerosol output. PSL nanospheres with the nominal sizes 22, 50, 75 and 100 nm were chosen to obtain well-defined, spherical, non-toxic and hydrophobic particles. Background particles, primarily smaller than 20 nm, were removed with a differential mobility analyzer (DMA, Model 3071, TSI GmbH, Aachen, Germany). The aerosol passes a Ni-63 neutralizer (design: Lund University, Lund, Sweden) before entering the DMA, to assure equilibrium charge. Downstream the DMA all particles have a single positive charge. Monodispersity of the aerosol was confirmed by characterization with a scanning mobility particle sizer (SMPS, design: Lund University, Lund, Sweden).

The monodisperse aerosol was led into a semi-flexible reservoir constructed of a 10 L rigid stainless steel tank and an antistatic 6 L rubber re-breathing bag. The monodisperse aerosol was diluted with particle-free air to a concentration of approximately 2000–6000 cm^−3^ at a flow-rate of 5–7 L/min. The aerosol in the reservoir was continuously substituted and the excess aerosol was removed through a filtered exhaust to assure a stable supply of test aerosol.

### Inhalation system

The subject breathed through a mouthpiece connected to a high-speed, computer controlled, four-way valve (as used in MasterScreen PFT, Viasys GmbH-Erich Jaeger, Hoechberg, Germany) that switches between particle free air, the test aerosol reservoir and the sample collector (Fig. 1). The breathing pattern was monitored at a resolution of 100 Hz by a flow meter (pneumotachograph, Type 2, Dr. Fenyves und Gut, Hechingen, Germany) connected to the mouthpiece. The valve was opening and closing the ports with a time precision around 100 ms and a volume accuracy of 80–150 mL. To reduce particle losses, tubing lengths were minimized and all parts, with the exception of the four-way valve, were made of conducting or anti-static materials. The flow meter, four-way valve and sample collector were enclosed in a temperature-controlled box (35 °C) to avoid condensation of water vapour. Temperature and relative humidity were monitored in the aerosol reservoir, the sample collector and at the particle detector. The sample collector consists of a 1 m long brass tube with dimensions adjusted to prevent mixing of the sample and to minimize flow resistance.

### Particle detection and analysis

**Figure 2:**
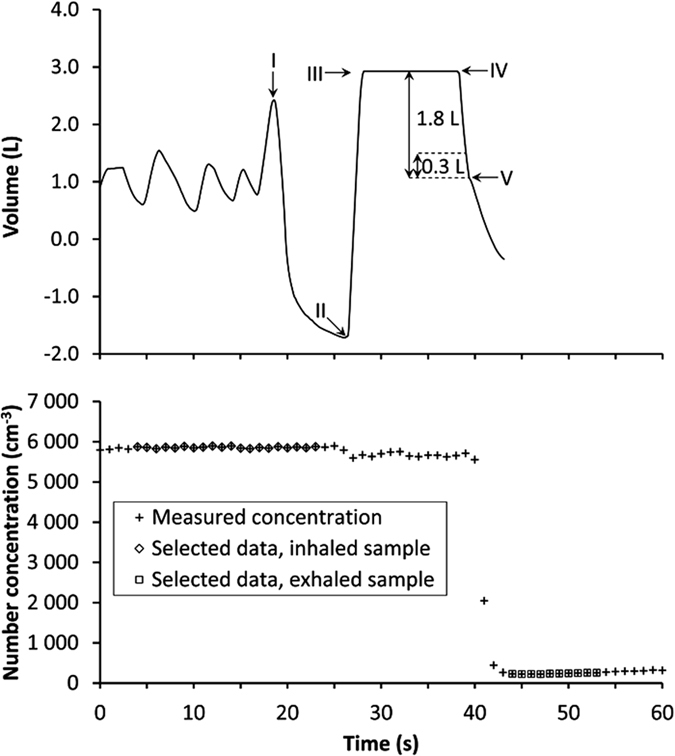
(Upper panel) Typical breathing pattern during measurements. From beginning of the measurement (min 30 s) to point (I), the subject breathed particle free air. Thereafter, the subject exhaled to residual volume. At point (II) the four-way valve switched and the subject inhaled particles from the aerosol reservoir. Between points (III) and (IV), all valves were closed and the subject held his breath. After a set period of time, at point (IV), the valve switched again and the subject exhaled into the sample collector. Once the determined sample volume was collected the valve to the sample collector was closed (V) and the subject exhaled to waste. (Lower panel) The measured particle number concentration during a typical measurement. The particle concentration in the aerosol reservoir was monitored until the exhaled sample was collected. Subsequently the exhaled aerosol concentration was measured. The particle recovery, R, was calculated from the mean values for the particle concentrations in inhaled and exhaled aerosols corrected for particle losses in the apparatus.

Aerosol was continuously sampled from either the aerosol reservoir or the sample collector for monitoring of the particle number concentration. A condensation particle counter (CPC, Model 3760, TSI Inc., Aachen, Germany) was used to measure the concentration at a frequency of 1 Hz and a flow of 1 L/min (Fig. 2). A mean value obtained over 20 s from the aerosol reservoir, and over 10 s from the sample collector, was used for inhaled and exhaled concentrations, respectively. Sample location was selected with a three-way computer controlled valve (Solenoid Valve, type 330, Bürkert, Ingelfingen, Germany). The relative humidity of the analyzed aerosol was reduced below 20% with a Nafion single-tube drier (MD-110-48S, Perma Pure, Toms River, NJ, USA). The pressure upstream the CPC was monitored (PasCal 100, Hoffrichter, Schwerin, Germany) to make sure that sampling flow was even. The sampling lines from the aerosol reservoir and the sample collector have equal lengths to avoid a systematic error from differences in particle losses. To confirm quality of the data, measurements were also performed with two alternative CPCs, a TSI 3010 (CPC, Model 3010, TSI Inc., Aachen, Germany) and an Airmodus (bCPC, Model a20, Airmodus Ltd, Helsinki, Finland). All CPCs are checked regularly and performed consistently in the setup and yielded similar results on reference subjects.

### Inhalation procedure

The breathing protocol mimics the procudure used for measurement of the diffusing capacity for carbon monoxide (D_L,CO_) and is thus known to be managable by the vast majority of the population without any need for prior training. Before the measurement, the position of the mouthpiece was adjusted to allow the subject to breathe comfortably. A nose clip was used to assure that the subject only breathed through the mouth. The operator and the subject could follow the subject’s breathing on a computer screen, which aided the subject to follow the breathing protocol accurately. The inhalation procedure is illustrated in Fig. 2 (upper panel).

During the first phase of measurement the subject breathes particle free air for a minimum of 30 s. Next, the subject performs an exhalation to residual volume (RV; Fig. 2, point I-II) followed by inhalation to total lung capacity (TLC; point II-III). At the transition between exhalation and inhalation (point II) the four-way valve opens the port to the aerosol reservoir. After complete inhalation all ports in the four-way valve are closed and the subject holds his breath for a set period of time. When the breath-holding period has elapsed, the port to the sample collector opens and the subject exhales normally. Once the predetermined volume has passed the sample collector, the valve closes and the breathing procedure is finished. The operator guides the subject through the procedure, and both operator and subject can follow the breathing pattern visually on a display. Data collection from a typical measurement are shown in Fig. 2 (lower panel). A sample volume of approximately 300 mL is drawn from the top of the sample collector, corresponding to air from 1.2–1.5 L volumetric lung depth (the dead space in the mouthpiece, flow meter and four-way valve, including anatomical dead space is estimated to 300 mL).

### Particle loss model

The particle recovery, *R*, is determined from the particle concentrations in the volumes for inhaled and exhaled aerosol (*C*_*reservoir*_, and *C*_*sample*_, respectively) with a correction for the penetration efficiency of particles in the mouthpiece, tubing and valves of the instrument expressed as *R*_*instrument*_:


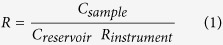


A semi-empirical model was constructed to correct for penetration efficiency of particles in the instrument. Because of the used particle size range, it was assumed that particle losses were due to diffusion. Diffusional losses deplete aerosol particle concentrations exponentially as a function of diffusion rate, container geometry and time. Loss of particles in the inhalation apparatus was measured by simulated breathing manoeuvres with a 3 L calibration syringe. The flow-rate was varied between 0.14 and 4.58 L/s. To minimize particle losses in the syringe the “breath-holding” period (III-IV in Fig. 2) was set to 200 ms. The measured particle recovery after passage through the system, expressed as *R*_*instrument*_, was fitted to an empirical equation describing measured particle losses in the apparatus as a function of passage time and particle size.


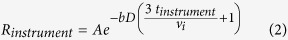


*D* is the particle size dependent diffusion coefficient, A and b are empirical fitting parameters. The passage time, *t*_*instrument*_, was derived from the individual breathing patterns when correcting *R* for individual particle losses during inhalation and exhalation, V_i_ is the end-inspiratory lung volume, also derived from the individual breathing pattern.

### Subjects

The system was evaluated for seven healthy, never-smoking volunteers, five males and two females, aged 20–34 years. Lung function, including measurement of vital capacity (VC), forced expiratory volume in one second (FEV_1_) and D_L,CO_, was measured according to current recommendations and the FEV_1_/VC ratio was calculated (MasterScreen PFT, Viasys GmbH-Erich Jaeger, Hoechberg, Germany)^12^. All subjects had normal spirometry and D_L,CO_. FEV_1_, VC, FEV_1_/VC and D_L,CO_ were 108 ± 10, 113 ± 11, 97 ± 3 and 91 ± 13% of predicted values^13^, respectively. The study was approved by the Regional Ethical Review Board at Lund and performed in accordance with the Declaration of Helsinki. Informed written consent was obtained from all subjects.

### Experimental design

The sensitivity of the instrument was characterized for particle size, particle concentration, and breath-holding time on seven young, healthy subjects. The particle sizes used were 22 nm, 50 nm, 75 nm and 100 nm. Tested breath-holding times were 3 s, 5 s, 7 s, 10 s, 15 s and 20 s. At least 3 measurements were carried out on each subject for each particle size and breath-holding time. To examine the sensitivity to alterations in flow-rate, measurements were performed on one subject with varying time for exhalation. During these measurements the inhalation of aerosol was made as a standard measurement, inhalation to FVC, but the exhalation flow rate was altered between 1.2–10.6 L/s which is covering the expected range of flow-rates for most subjects. To examine the sensitivity to inhaled particle concentration, measurements were carried out on one subject with particle concentrations varied from 1000 to 10 000 cm^−3^. The instrument was also characterized for inter-subject variability by comparing the measurements for the seven subjects. The stability over time was also evaluated from repeated measurements over a one year period on one subject. The inhaled particle concentration varied between 3000 and 6000 cm^−3^ for different particle sizes and measurement occasions during the experiments.

### Model calculation of recovery

The accuracy of the measurements was evaluated by comparison with estimated results from the Multiple-Path Particle Dosimetry model (MPPD V2.1, Applied Research Associates Inc., Albuquerque, N.M, US, 2009). The model is constructed for tidal breathing patterns at constant exposure and could thus not fully replicate the procedure used in the measurements. A breathing pattern with 2.5 s inhalation and expiration times, a 10 s breath hold and tidal volume of 4500 mL was used. The Yeh-Shum 5-lobe lung model^14^ was chosen for the calculations.

## Results

### Sensitivity to particle size, breath-holding time, and particle concentration

**Figure 3:**
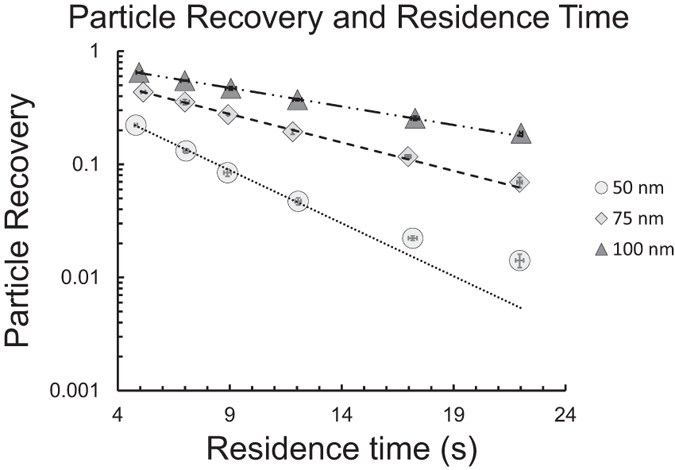
The recovery, R, for three particle sizes at varying breath-holding times. Data are for one subject. Standard deviations show variations between three or more measurements (most of the error bars are too small to be visible on this scale).

Particle recovery, *R*, for various particle sizes and breath-holding times for one subject is shown in Fig. 3. As expected from the theory of Brownian diffusion, *R* decreased with increasing breath-holding time and decreasing particle size. For 22 nm particles *R* was close to zero and therefore the effects of breath-holding time were difficult to observe.

The precision of the measured *R* was assessed from the variability during repeated measurements. The standard deviation of *R* for repeated measurements with the instrument was typically within 0.002–0.008, which is small compared to the total variation of *R* that ranges between 0–0.65 for the seven subjects. Further, the stability over time was evaluated for one subject inhaling 50 nm particles with a 10 s breath-hold at 15 different occasions during a one year period. *R* for these measurements was 0.0538 ± 0.003, which indicates a high consistency of the data. It was also verified that variations in particle concentration (within concentration span from 1000–10000 cm^−3^) in the reservoir for inhaled air did not alter the measured *R* as long as the concentration was stable during the measurement period.

### Sensitivity to aerosol flow-rate

**Figure 4:**
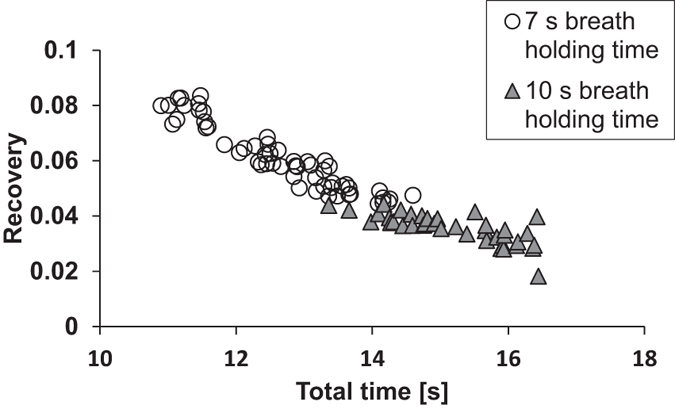
Observed recovery for 50 nm particles with different flow rates (1.2–10.6 L/s) at 7 s and 10 s breath-holding times, plotted as a function of total residence time. The data shows that there is correlation between recovery and total residence time (Pearson’s correlation coefficient = 0.94).

**Figure 5:**
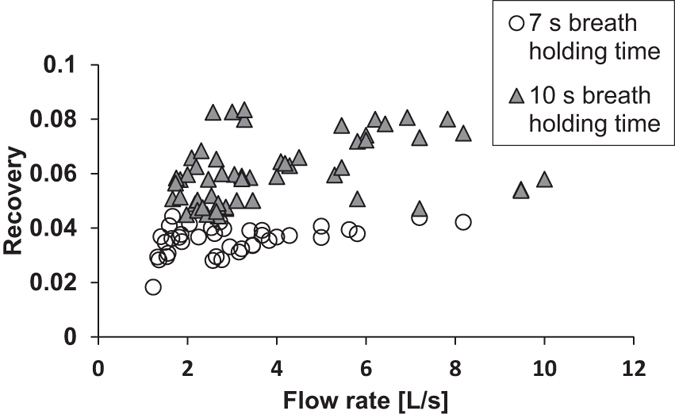
Observed recovery for 50 nm particles at different flow-rates (1.2–10.6 L/s) with 7 s and 10 s breath-holding times (the same data as shown in Fig. 4), plotted as a function of flow-rate. No correlation was found between flow-rate and recovery.

Measurements were made on the same subject at different flow-rates for two breath-holding times (Figs 4 and 5). The residence time in the lungs was defined as the time from when half of the test aerosol was inhaled to the midpoint time of the exhaled sample. As seen in Figs 4 and 5, the main determinant of the observed recovery, *R*, is the residence time in the lungs (Pearson´s correlation coefficient = 0.94). Flow-rate does not strongly influence the results.

### Inter-subject variability and comparison with modelling

**Figure 6:**
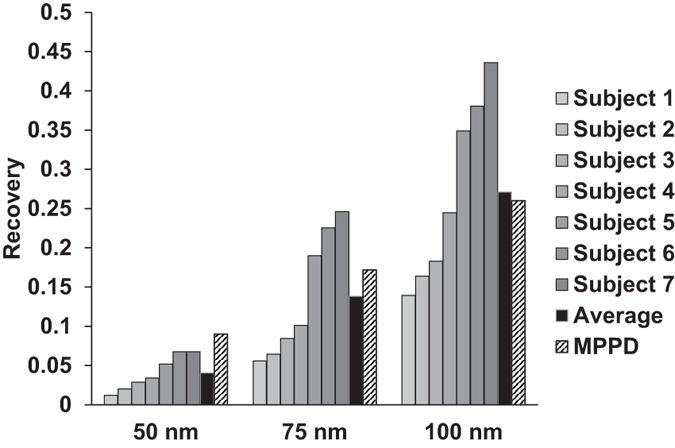
Particle recovery (R) for seven subjects, normalized to 10 s residence time in the lung, compared to calculation with the MPPD model. The variability between the subjects is 26–50 times larger than the measurement precision.

The measured particle recovery for seven subjects breathing 50, 75 and 100 nm particles are shown in Fig. 6. The recovery was time normalized to 10 s residence time in the lungs by curve fitting. Values ranged between 0.012 and 0.068 for 50 nm particles, 0.06–0.24, for 75 nm particles and 0.14–0.44 for 100 nm particles. Standard deviations were on average 0.0022, 0.0080 and 0.0088 for the three particle sizes, respectively, at 10 s breath holding time. Thus, although the investigated group was homogenous (i.e. healthy and aged 20–35 yrs), the inter-subject variability was 26–50 times larger than the measurement precision, evaluated as the standard deviation for repeated measurements. Some subjects had a slightly higher standard deviation for repeated measurements, up to maximum 0.011. This was probably due to inconsistency when following the breathing protocol. As shown in Fig. 6, the *R* values calculated with the MPPD model were in general agreement with the measurements. However, it was not possible to adjust the MPPD model to fully mimic the measurements and therefore the comparison could only provide an indication on the accuracy of the experiments, compared to the average for the 7 subjects.

### Particle loss model

**Figure 7:**
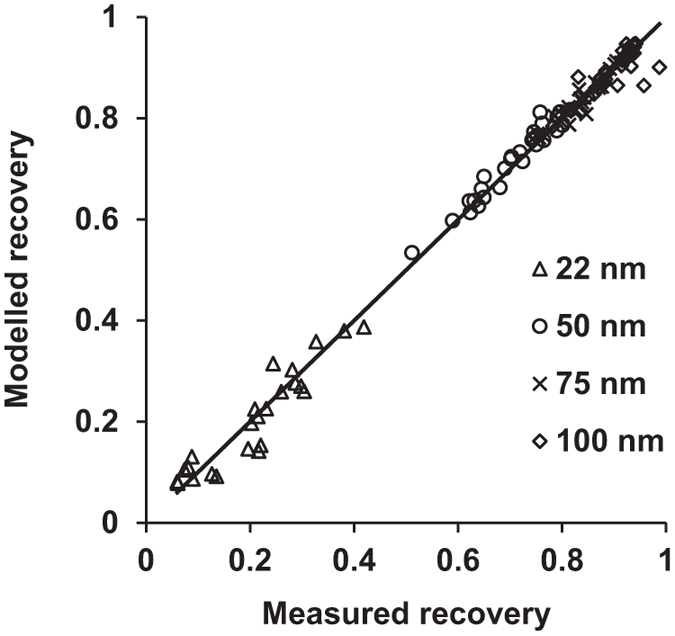
Observed and modelled values for penetration efficiencies, i.e. recovery, in the inhalation system for various particle sizes and flow rates.

The measured and modelled particle penetration efficiencies through the instrument, reported as recovery, *R*_*instrument*_, are illustrated in Fig. 7. *R*_*instrument*_ varied from 0.948 for 100 nm particles at short residence times to 0.0781 for 22 nm particles with long residence times. Recovery measurements for 22 nm particles are uncertain, since losses in the instrument at residence times exceeding a few seconds are above 90%.

The best fit of the loss model (Equation 2) was obtained with a value of 1.00 for constant A and 2.63·10^7^ for the exponent b. The Pearson correlation coefficient between the observed data and theoretical values calculated from the particle loss model was 0.999.

## Discussion

As shown by the results, the developed instrument measures the recovery of inhaled nanoparticles over a single breath with high sensitivity to changes in particle size, breath-holding time and subject characteristics. The recovery of inhaled nanoparticles was measured for a wide range of particle sizes and different breath holding times in a small group of healthy volunteers. Even though the subjects were a homogeneous group, the inter-subject variability was significantly higher than the intra-subject variability, and the results compared well to estimations from the MPPD model.

The experimental setup was designed considering the critical criteria and challenges stated in the introduction and elsewhere^1^. A stable concentration of aerosol in the inhalation reservoir was obtained by a flow through system. The aerosol reservoir was dimensioned to hold enough aerosol for breathing manoeuvres at forced vital capacity (FVC) and to dampen out small temporary concentration fluctuations, but still to provide fast mixing of the aerosol and aerosol exchange rates on the time scales of minutes. By continuously monitoring the aerosol from the aerosol reservoir, the reference for inhaled particle concentration could be assessed accurately.

In previous studies a number of different aerosol sources have been used, ranging from ambient air to spark discharge aerosols, oil droplets or even tobacco smoke^5^,^6^,^8^,^15^,^16^. A few experiments have also been performed with monodisperse aerosols^3^,^17^,^18^. The electrospray has the advantages of a low, well-defined background that can be easily removed in the DMA, which also removes highly charged particles. Downstream the DMA the particles carry a single positive charge, which is unlikely to influence deposition. The particles used in the system are hydrophobic without hygroscopic residues. A limitation with the approach is the complexity of the electrospray and laborious maintenance for consistent performance.

The particle concentration in the inhaled and exhaled aerosol was measured with a condensation particle counter (CPC). There is a limited choice of technologies to detect and quantify airborne nanoparticles, especially if a fast response time is of importance. Previous reported studies have used several different technologies to detect and quantify nanoparticles, e.g. SMPS-systems^5^,^9^,^10^,^19^,^20^,^21^,^22^, gamma cameras^23^,^24^,^25^,^26^, α- or β-counters^27^,^28^,^29^, flame photo detection^30^, light scattering devices^2^,^15^,^16^,^31^,^32^,^33^, tapered element oscillating microbalance (TEOM)^5^,^21^, filter collection^27^,^28^,^29^, impingers^34^,^35^ and impactors^34^,^35^. These techniques have various challenges: α- or β-counters or gamma cameras require radiolabelled particles; light scattering devices are not reliable for particles smaller than approximately 400 nm; filter collection, impingers and impactors have to be analysed offline. A TEOM provides a measure of total particle mass, but has poor time resolution and is sensitive to pressure fluctuations, which are hard to avoid when measuring from an inhalation system. A complete SMPS system is redundant as the aerosol is monodisperse and only the number concentration is of interest. When taking these factors into consideration, basically two alternatives remain for nanoparticles: condensation particle counters (CPC) or Faraday cup electrometers. Only one previous study of lung deposition of nanoparticles reports using an electrometer^36^. Electrometers have the advantages of simplicity and robustness but are less sensitive at low particle concentrations than condensation particle counters.

The breathing protocol used in this study is basically the same as used in the measurement of D_L,CO_^12^. A modified version of the breathing protocol has previously been used in lung deposition studies, more specifically in the “single-breath” and the “particle concentration” versions of aerosol-derived airway morphometry^37^,^38^ (ADAM). It has also been used in one study to determine particle deposition of nanoparticles, but with a bolus of radiolabelled particles^7^. The first exhalation to RV followed by inhalation to TLC minimizes the effect of mixing and dilution with residual air in the lungs. The rapid reduction in linear flow velocity in the distal lung and the breath holding time allows the nanoparticles to move by diffusion with minimal convective motions. Thus, the measured particle recovery is expected to correlate with the diffusion distances in the distal lung in their most inflated state.

It was concluded that the particle recovery declined exponentially with residence time in the lung, as expected from the theory of Brownian motion and previous studies^18^,^36^,^39^. However, comparison to previous work is not trivial. The available data for studies using nanoparticles are limited, and especially studies with a well-defined and varied residence time in the lung are missing. A study by Blanchard and Willeke^36^ reports data for measurements on one individual with different breathing periods to illustrate the influence of residence time in the lung. They used hygroscopic saline particles that (when they are at equilibrium with the moist air in the lung) have a size around 100 nm. The reported particle deposition is in the range of the data observed in this study (*R* ≈ 0.60–0.48 at 3–6 s estimated residence time), although the methodology differs on several important points. More studies with data for particle deposition during varied residence times are only available for larger, micron-sized particles.

The largest measurement uncertainties were found for the shortest (3 s) and longest (15–20 s) breath-holding times. The uncertainties at short residence times were expected, as the breath-holding time is short compared to the inhalation/exhalation procedure. At longer residence times the exhaled particle concentration is low, and even a minor contribution of particles from larger airspaces will have a large impact on the result. However, for all breath-holding times the repeatability of the results was within 7.4%, 5.7% and 2.1% of the measured *R* for 50, 75 and 100 nm particles respectively (Fig. 3).

The measured recovery for the seven healthy subjects showed that the instrument had a precision significantly higher than the inter-subject individual variation. This indicates that the method could be suitable for investigating individual variations in lung deposition and morphology, which have been observed in previous studies^11^,^40^,^41^.

There are several putative applications of the developed instrument: investigation of effects of inhaled nanoparticles at various regions of the lung, precise nanoparticle drug delivery, exposure assessment in toxicological studies and for assessment of individual variations in lung morphology. For instance, with minor modifications to the instrument software, aerosol can be delivered as boluses to specific volumetric lung depths. As the method is fast and uncomplicated (a measurement takes less than a minute and the breathing manoeuvre is known to be manageable also for patients with severe respiratory disease) it is suitable for screening studies to investigate distributions on large populations. This could contribute to a better understanding of questions related, for instance, to individual variations of lung morphology, where new methods are of high demand^42^. Targeting the alveolar region of the lung is an attractive route for administration of peptide based pharmaceuticals such as insulin and has been suggested as a possible route for gene therapy and administration of monoclonal antibodies^43^,^44^,^45^, avoiding the problem of mucociliary clearance followed by second pass metabolism of inhaled particles^46^. Using the setup to deliver pharmaceuticals is primarily of interest for potent substances as it is difficult to deliver high mass concentrations by nanoparticles.

An application of special interest may be as a technique for assessment of pathological changes in lung anatomy, similar to previously developed aerosol-based techniques such as ADAM (e.g. refs 37,38,47, 48, 49, 50, 51, 52, 53, 54). The here proposed technique, where nanoparticles rather than micron-sized particles are used, will further be referred to as Airspace Dimension Assessment (AiDA). AiDA has several similarities to ADAM, but differs regarding deposition mechanisms and technology. Particles in the size range 800–1000 nm are mainly deposited by sedimentation and inertial impaction. The particle size used in the ADAM techniques and in the aerosol bolus dispersion techniques is deliberately chosen to maximize the effect of sedimentation and to minimize the effect of diffusion^55^. The theoretical models used to analyse ADAM measurements assume that all particles are deposited exclusively by sedimentation^55^, although this is a simplification and as diffusion and, for moderate to high flow rates also impaction, will contribute. In contrast, diffusion is much more dominant for the AiDA technique and no other deposition mechanism need to be taken into account.

A substantial advantage with AiDA is the possibility to use comparatively high airflows during inhalation and exhalation. In ADAM, an airflow of 250 mL/s is typical in order to achieve repeatable and minimal particle losses^54^. This can be challenging for untrained subjects or patients with respiratory disease. It also makes measurements at full lung inflation problematic. As seen in Figs 4 and 5, the deposition of nanoparticles is almost completely independent of flow-rates during inhalation and exhalation with flows up to 10 L/s. This simplifies the breathing manoeuvre and enables measurements at full lung inflation.

The proposed diagnostic application of the technique, AiDA, also has several similarities with the measurement of diffusion capacity of the lungs for carbon monoxide, (D_L,CO_)^56^,^57^. D_L,CO_ is a clinical standard technique with sensitivity for emphysema, but with less specificity compared to computed tomography and magnetic resonance imaging^56^. Similarly to D_L,CO_, the particle recovery, *R*, is measured for air from a volumetric lung depth of about 1.5 L. As described earlier, particle recovery is expected to correlate with the diffusion distances of the distal lung units. AiDA might also reflect distal air space enlargement more specifically than D_L,CO_, as AiDA is expected to reflect the diffusion distances within the air spaces only, whereas D_L,CO_ depends also on a number of other factors such as the properties of the blood-gas barrier and the capillary blood volume^58^. Judging from the limited number of measurements in this study, the precision of AiDA is comparable, or better, than that of D_L,CO_. The coefficient of variation of repeated within-session measurements of D_L,CO_ is approximately 5%, i.e. similar to AiDA, whereas the long-term reproducibility is about 9% for D_L,CO_ but about 5% for AiDA in this study^12^.

In conclusion, the presented instrument provides data for individual measurements of lung deposition of nanoparticles with size-resolution and time resolution on human subjects with precision on par with, or better than, in previous studies. The method has several advantages: it is fast, has high precision, yields highly reproducible results and can target specific volumetric lung depths. Possible applications are as a method to investigate the effects of inhaled nanoparticles at various regions of the lung, as a device for precise nanoparticle drug delivery, for exposures assessment in toxicological studies, or as a diagnostic tool for the lungs with possibility to monitor the progress of disease or effect of applied therapy.

## Additional Information

**How to cite this article**: Jakobsson, J. K. F. *et al*. A new method for measuring lung deposition efficiency of airborne nanoparticles in a single breath. *Sci. Rep.* **6**, 36147; doi: 10.1038/srep36147 (2016).

**Publisher’s note**: Springer Nature remains neutral with regard to jurisdictional claims in published maps and institutional affiliations.
